# Pacemaker Infection and Parvimonas micra-Induced Prosthetic Aortic Valve Endocarditis With Periannular Abscess: A Case Report

**DOI:** 10.7759/cureus.93749

**Published:** 2025-10-02

**Authors:** Monique Boukobza, Pierre Aubry, Jean-Pierre Laissy

**Affiliations:** 1 Radiology, Public Assistance-Paris Hospitals, Bichat-Claude Bernard Hospital, Paris, FRA; 2 Cardiology, Public Assistance-Paris Hospitals, Bichat-Claude Bernard Hospital, Paris, FRA

**Keywords:** infectious endocarditis, mechanical valve, pacemaker, parvimonas micra, periannular abscess, prosthetic aortic valve

## Abstract

A 59-year-old man was admitted for a prosthetic aortic valve (AV)infectious endocarditis (IE), attributed to the pacemaker and complicated by a circumferential periaortic circulating abscess, a severe aortic periprosthetic leak, and left-sided heart failure. Blood cultures were negative on hospital day 3. He underwent an emergent AV biological valve replacement, and 16S rRNA bacterial gene sequencing performed on AV tissue identified the microorganism as *Parvimonas micra*. We reviewed the 11 cases of *Parvimonas micra *IE previously described. Some features seem to be abnormally frequent in this context: history of prosthetic valve or cardiac device, pacemaker infection, periannular abscess, and chronic dental infection.

## Introduction

Previously known as *Peptostreptococcus micros*, *Parvimonas micra* was attributed to the *Micromonas* genus (*Micromonas micros*) and then assigned to the *Parvimonas* genus. *Parvimonas micra* is a gram-positive anaerobic coccus, commensal to the normal oral flora in humans, and the only member of *Parvimonas* genus. *Parvimonas micra* (*P. micra*) is a fastidious, slow-growing bacterium that is difficult to isolate and that needs enriched culture media and anaerobic atmospheres. However, in recent years, the introduction of new technology for the identification of microorganisms, such as matrix-assisted laser desorption/ionization time-of-flight (MALDI-TOF) and 16S rRNA sequencing, has led to increased diagnosis of *P. micra*. *P. micra* has been implicated in oral cavity infections [1], mainly mucositis, sialadenitis, and parotitis, but has also been described as the causal agent of the gastrointestinal tract, oropharyngeal, intraabdominal region (abscesses), respiratory (including empyema), spondylodiscitis, osteomyelitis, native and prosthetic joints, meningitis, brain abscesses, isolated or associated with other abscesses, and purulent pericarditis [2].

The link between poor dental hygiene, recent dental infections/procedures, and *P. micra* infection is well established. There are other risk factors, including immunosuppression/immunodeficiency. *P. micra* has been associated with comorbidities such as diabetes mellitus, brain tumor, chronic B hepatitis, heart valve prosthesis, transplantation, cancer, hematologic malignant disease, chemotherapy, and steroid treatment. When present, they should raise suspicion for infection due to this pathogen. Some reports suggested an association between colorectal cancer and *P. micra* [3]; thus, it is important to explore these patients widely (whole-body CT, positron emission tomography (PET)/CT), including gastrointestinal tract. However, cases with unknown portal of entry and without any past medical history have been reported.

Watanabe et al. [4] found that *P. micra* bacteremia was part of polymicrobial bacteremia in about 45% of their 25 cases, mostly IE on native valve. The same authors, in their literature review based on 27 cases, observed that *P. micra* bacteremia was frequently associated with spondylodiscitis (29.6%), oropharyngeal infection (25.9%), intra-abdominal abscess (14.8%), infective endocarditis (11.1%), septic pulmonary emboli (11.1%), and gastrointestinal tract (GIT) infection (11.1%).

Despite causing severe infections, *P. micra* has shown wide antimicrobial susceptibility. In recent years (2005-2015), sporadic cases of *P. micra* infectious endocarditis (IE) have surged. *P. micra* is increasingly recognized as a pathogen in IE, particularly in those with prosthetic cardiac valves. We report a case of *P. micra* IE in a man with a long cardiac history, and we review the 11 previously reported cases, trying to identify the possible common background and comorbidities in this rare IE.

## Case presentation

An obese 59-year-old man (116 kg/185 cm, body mass index (BMI): 33.9 kg/m^2^), with a history of hypertension, dyslipidemia, and alcohol consumption, was admitted with a four-month history of left-sided heart failure symptoms, i.e., worsening shortness of breath on minimal exertion. The patient’s cardiac history included a mechanical (Cardiomedics 25, CARBO-SEAL AP-025, CORCYM S.r.l., Saluggia, Italy) aortic valve (AV) replacement for a bicuspid calcified valve and severe stenosis 10 years ago. Consequently, he underwent a pacemaker (PM) implantation for persistent complete atrioventricular block (AVB). Five years ago, he was treated with one stent on the first segment and two stents on the second segment of the left anterior descending artery (LAD), in a single procedure, for non-ST elevation myocardial infarction (NSTEMI).

For the past one month, he complained, almost daily, of mid-thoracic, squeezing pain, radiating to the jaw and left shoulder. Hospitalized in a peripheral hospital, the diagnosis was acute coronary syndrome (ACS) with ST elevation myocardial infarction (STEMI) and reduced ejection fraction (troponin peak: 5,800 ng/l then 1,600 ng/l at discharge). Coronary angiography showed no significant coronary disease. The diagnosis of ACS was not revised.

**Figure 1:**
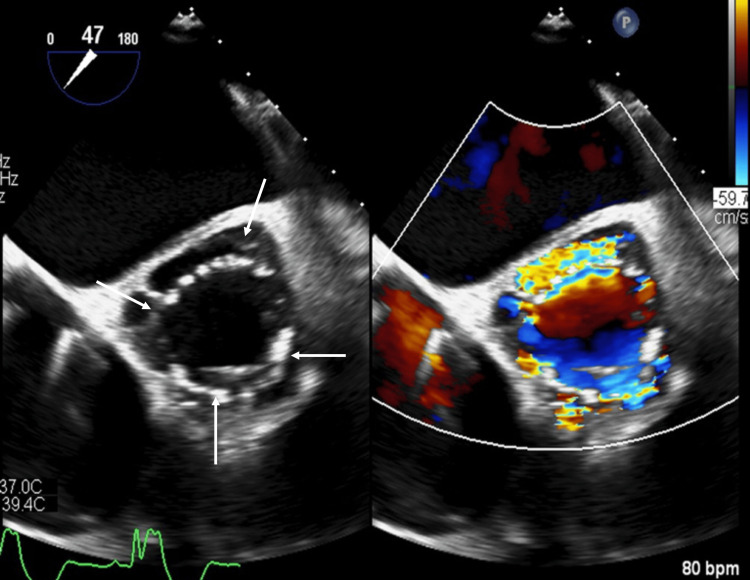
Transesophageal echocardiography shows contrast enhancement (arrows) around the aortic bioprosthesis similar to cardiac vascular enhancement on CTA, confirming the perivalvular abscess with aortic lumen communication turbulent flow inside on color Doppler images. CTA: CT angiography.

**Figure 2:**
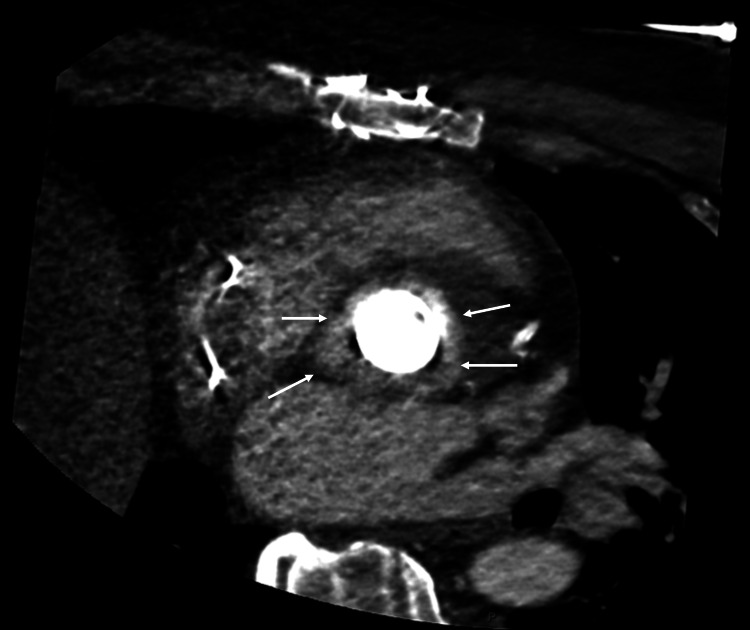
On CT angiography (CTA), contrast enhancement (arrows) around the aortic bioprosthesis is like cardiac and vascular enhancement of the ascending aorta, confirming the perivalvular abscess with aortic lumen communication.

**Figure 3:**
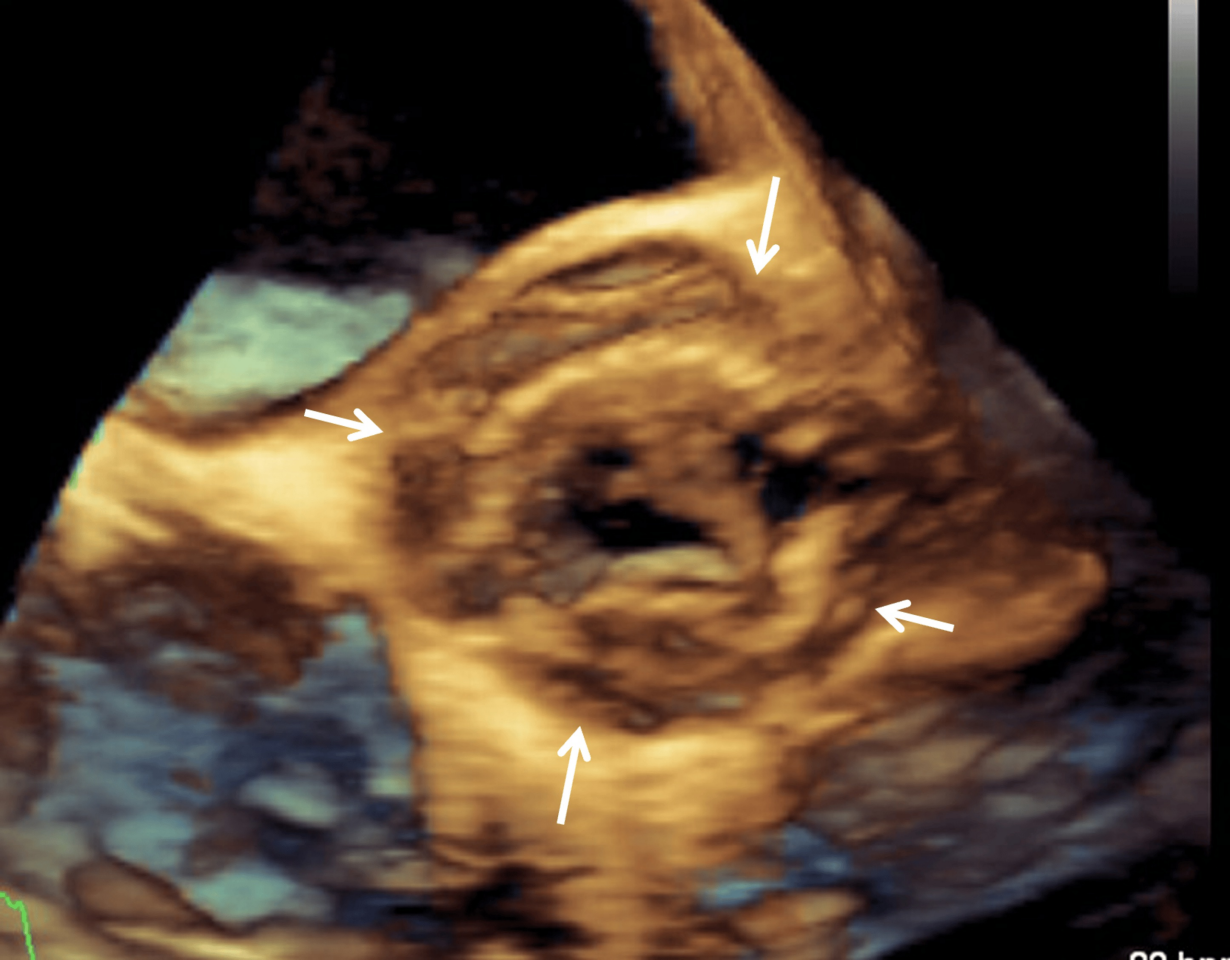
Transesophageal echocardiography shows the aortic bioprosthesis surrounded by a periannular abscess on 3D images (arrows).

At admission, he was febrile and complained of midthoracic pain. Transthoracic and transesophageal echocardiogram (TTE/TEE) showed a circumferential periaortic circulating abscess (Figures 1-3), severe aortic periprosthetic leak, and dilation of the left ventricle with marked hypocontractility. A diagnosis of IE was made, and the origin of IE was attributed to a PM infection. A treatment with Bactroban (nasal route) was started upon arrival and switched three days later to vancomycin/gentamicin.

Whole-body CT angiography (CTA) revealed splenic infarcts and brain CTA showed multiple infarcts, both supra- and infratentorial. The cranio-cervical vessels were unremarkable. Blood cultures were negative; at hospital day (HD) 3, a rare germ test was performed (molecular diagnostic panel). At HD 3, the patient underwent an emergent AV biological valve replacement redux, a mitral-aortic trigone patch for abscess extension, and the pacemaker was removed and replaced by an epicardial pacing system. Later, 16S rRNA bacterial gene sequencing (broad-range polymerase chain reaction (PCR) and sequencing) performed on aortic valve tissue identified the microorganism as *P. micra*. At one-year follow-up, the patient has been stable, without infectious or cardiovascular recurrence.

## Discussion

*P. micra* IE is a rare entity. Only 11 case reports involving 11 patients with *Parvimonas micra* IE have been published to date [2,5-14] and are summarized in Table 1 (systematic literature review, January 2005-June 2025), along with our case (12 *Parvimonas micra* IE patients in total). The occurrence of reported cases since the first description in 2015 by Gomez et al. [5] is increasing over a 10-year period: four reported cases between 2015 and 2019 and eight between 2020 and 2024.

**Table 1:** Characteristics and treatment data of published cases of Parvimonas micra infectious endocarditis. Ref: Reference; yr(s): year(s); IE: infectious endocarditis; M: male; AV: aortic valve; MV: mitral valve; VR: valve replacement; NA: not available; NP: not performed; F: female; TV: tricuspid valve; PM: pacemaker; DM: diabetes mellitus; AF: atrial fibrillation; AVB: auriculoventricular block; 4M: 4 months.

Case	Author/Year/Ref	Age (yrs)/ Sex	Risk factors for IE	Valve/Type	Echocardiographic findings	Associated lesions	Antimicrobial therapy	Cardiac surgery	Outcome (months of follow-up)
1	Gomez et al., 2015 [5]	71/M	None	AV, MV/Native	Perivalvular abscess (15 mm)	-	Vancomycin + Nafcillin + Gentamicin → Ampicillin/Sulbactam	AV, MV/VR	Favorable (12)
2	Iwasaki et al.,2017 [6]	78/M	Laryngeal cancer/total laryngectomy, poor oral hygiene	AV/Native	NA	Pulmonary infarct, lung abscess→ drainage	Ampicillin/Sulbactam → Clindamycin	NP	NA
3	Maki et al., 2017 [7]	48/F	Tooth extraction, PM	TV/Native, PM leads	Vegetation, septic pulmonary embolism, *Fusobacterium nucleatum* (bloodstream coinfection)	-	Ampicillin/Sulbactam → +Gentamicin → Ampicillin → + Metronidazole	NP	NA
4	Hoet al., 2018 [8]	42/M	Tooth extraction, mechanical MV/VR, DM	MV, mechanical	MV/2mobile vegetations, severe regurgitation	-	Ceftriaxone + Vancomycin → Penicillin G	Repair of the mitral paravalvular leak	NA
5	Garcia-Hitaet al., 2020 [9]	63/F	PM, subependymoma, mechanical MV/VR (30 yrs)	MV, mechanical	Vegetations (8 mm)	-	Ceftriaxone + Rifampicin	NP	Favorable (12)
6	Morinaga et al., 2021 [2]	70/M	Decayed tooth, purulent, pericarditis → abscess/pericardial space	Native valves	Mobile vegetation (19.2x9.2 mm), right atrium	-	Ampicillin + Sulbactam	Removal of the right atrium, vegetation and infected wall	Favorable (6)
7	Suzukiet al., 2021 [10]	82/M	Poor oral hygiene, PM	TV/Native, PM leads	Vegetation on PM leads/TV	PM infection	Ceftriaxone → Ampicillin + Gentamicin → Ampicillin	Removal of permanent PM (PM infection)	Favorable (12)
8	Callegariet al., 2023 [11] Case 1	73/F	Immunodepression infliximab	MV/Native	Vegetation (10 mm) perivalvular abscess	Ischemic strokes	Penicillin (4M x 4/day)	NP	NA
9	Azizet al., 2024 [12]	60/M	AF, mechanical MV/VR	MV, mechanical	Vegetations (9 mm)	Multiple ischemic strokes	Benzylpenicillin (1,200 mgx6)	NP	Survived
10	Fuet al., 2024 [13]	68/M	Permanent PM (26 yrs), mechanical MV/AV/VR (26 yrs), biological TV (1 yr)	Mechanical AV, mechanical MV	AV regurgitation TV, vegetation, thickened TV, bioprosthesis	-	Post-operative diagnosis of *Parvimonas micra* IE: Imipenem→ Piperacillin-Tazobactam	Emergent TV, bioprosthesis/AV, mechanical VR, PM lead penetrated the prosthetic TV	NA
11	Nakataet al., 2024 [14]	78/F	Colorectal cancer	MV/Native	Mobile vegetations, MV regurgitation	Stroke	NA	NP	Colorectal cancer excision, survived
12	Present case, 2025	59/M	AV, mechanical VR (12 yrs) (bicuspid AV, calcified stenosis), PM (AVB) (12 yrs)	AV, mechanical	Periannular abscess	Multiple ischemic strokes, splenic infarcts, PM infection	Bactroban →Vancomycin Gentamicin	Hospital day 3 AV/VR/bioprosthesis, mitral-aortic trigone patch/PM removal, epicardial pacing system	Favorable (12)

Among the 12 *Parvimonas micra* IE patients, including our case and the 11 reported in the literature, eight were male, and the same percentage of patients were over 60 years of age. Regarding comorbidities, five (41.7%) patients had a history of dental procedures, periodontitis, caries, or poor oral hygiene, which are the main associated comorbidities in *P. micra* infection, and a dental origin was confirmed in three of five patients.

Three (25%) had a history of neoplasm, and one (8.3%) was under immunosuppressive therapy. Only one patient presented with diabetes mellitus, and one (case 3, Table 1) had a bloodstream coinfection by *Fusobacterium nucleatum*. The main risk factors for *P. micra* infection are dental procedures, periodontitis, tooth extraction, and abscesses or caries.

Regarding the risk factors for IE, a total of seven patients (58.3%) had a prosthetic device involving the heart. Five patients had prosthetic valves (cases 4, 5, 9, 10, and our case), three of whom also had PM (cases 5, 10, and our case), and two had only PM (cases 3 and 7). Late (>12 months) prosthetic valve endocarditis usually has a distribution of microorganisms like that observed in native valve infection, contrary to the cases of *P. micra* IE collected here.

Late prosthetic valve endocarditis (PVE) may also be caused by *Cutibacterium acnes*, a low-virulent anaerobic bacterium [15]. Molecular techniques, including polymerase chain reaction (PCR) and metagenomic next-generation sequencing, have been useful in the etiologic diagnosis of PVE. In addition, in one of these patients (case 9), the PM lead penetrated the prosthetic TV, without regurgitation or abscess which represent potential complications.

Regarding the valvular complications, perivalvular abscess was observed in three patients (25%; cases 1, 8 (Table 1), and our case), and regurgitation in three (25%; cases 4, 10, and 11). Based on our review of the literature, the mitral valve is most often affected (n=6, 50%). The case by Morinaga et al. [2] is very unusual in that it concerns a patient with no remarkable medical history, suffering from decayed teeth who presented a purulent pericarditis. *P. micra* was detected at HD 11 on both blood and pericardial fluid.

Three days later, CT diagnosed an abscess in the pericardial space and emergency surgery confirmed the presence of a large and mobile intraluminal vegetation and perforation of the right atrium with communication to the abscess cavity. Surgery and antibiotic therapy allowed a favorable outcome (case 6). Complications were lung abscess requiring drainage (n=1; case 2) and embolic events in five, four of which involved cerebral ischemia on brain imaging (cases 8, 9, 11, and our case). Six patients (50%) required cardiac surgery in addition to antibiotics for complete recovery. There was no in-hospital mortality.

Our case is remarkable for the association of a prosthetic valve and a pacemaker and a periannular abscess. Even if a pacemaker infection was suspected as the portal of entry, there was no bacteriological confirmation. Moreover, ENT and dental examination as well as colonoscopy did not provide any feature of local infection. It should be emphasized that, given the current literature, each of these features seems to form part of the background of *P. micra *IE patients, but none of the reported cases has such a severe cardiac background. In addition, tricuspid valve *P. micra* IE, with vegetation, has only been reported once [10].

Nevertheless, cardiac surgery was without complications, and there was no antibiotic resistance. No dental portal of entry was found, and we know that not every patient with a *P. micra* infection has a dental portal entry. Concerning medical treatment, most studies suggest that *P. micra* has shown wide sensitivity to multiple antibiotics used against anaerobes, such as lactams, clindamycin, and metronidazole, although isolates resistant to clindamycin and vancomycin have been found [16]. The follow-up, reported in only seven cases, showed a favorable outcome (i.e., no recurrence) at 12 months (clinical follow-up: cases 1, 7, 9, 11, and our case; radiological follow-up: cases 5, 6, and our case).

Thus, despite causing severe infections, literature findings show that *P. micra* IE patients, as other *P. micra* infections, might have a good prognosis following appropriate treatment. Due to the small number of *P. micra* IE cases reported, no conclusions can be drawn, hence the need for a multicenter case series to inform trends. Nonetheless, some clinical-epidemiological characteristics seem more common: the incidence of previous dental procedure or poor oral hygiene, mechanical prosthetic cardiac valves (n=5, 41.7%; MV: 3; AV: 1; MV/AV: 1), PM (n=5, 41.7%), and oncological history are more common than overall IEs reported in great European and US series [17,18]. In our series of 520 consecutive patients with definite IE, 21% of patients had prosthetic valves and 13.4% were PM wearers and 8% had an oncologic history, with active malignancy in 6% of cases.

## Conclusions

Several points of this rare pathology must be emphasized. It should also be noted that it is difficult to draw definitive conclusions from a limited number of cases. First, the predisposing conditions, especially in our patient who had a PM with a device-related infection, were considered important risk factors. Second, despite a long cardiac history and a periannular abscess, the outcome of our patient was favorable.

Based on the 12 cases reported to date, including ours, some features seem to be abnormally frequent in this context: history of prosthetic valve or cardiac device, pacemaker infection, chronic dental infection, and oncologic history. *P. micra* IE is often complicated by periannular abscess in three of 12 (25%) cases in our literature review. Additionally, the introduction of 16S rRNA bacterial gene sequencing should facilitate the diagnosis of *P. micra* IE and is especially helpful in culture-negative IE. Patients with *P. micra* IE could have a good prognosis, even with complicated background (i.e., prosthetic valve, multiple comorbidities), following appropriate treatment when guided by antibiogram sensitivity.
